# Clinical characteristics and follow-up of a newborn with Dubin–Johnson Syndrome: A clinical case report

**DOI:** 10.1097/MD.0000000000036991

**Published:** 2024-01-26

**Authors:** Yanmin Zhang, Wei Zuo, Wei Gao

**Affiliations:** a Department of Neonatal Surgery, Anhui Provincial Children's Hospital (Anhui Hospital, Pediatric Hospital of Fudan University), Hefei, Anhui, China.

**Keywords:** bilirubin, compound heterozygous mutations, DJS, exome sequencing, follow-up, liver enzyme, neonatal liver disorder

## Abstract

**Background:**

Dubin–Johnson syndrome (DJS) is a rare autosomal recessive liver disorder, characterized by conjugated hyperbilirubinemia. This case report investigates the clinical characteristics and longitudinal outcomes of a neonate diagnosed with DJS.

**Methods:**

A newborn presented with elevated bilirubin levels and abnormal liver enzyme readings. Comprehensive genetic evaluation was conducted, which included peripheral blood sample collection from the infant and both parents after obtaining informed consent and high-throughput trio exome sequencing was performed. The genetic analysis revealed 2 significant mutations in the *ABCC2* gene on chromosome 10: the insertion mutation *c.4237(exon30)_c.4238(exon30)ins CT*, inherited from the father, and the missense mutation *c.517(exon5)G > A*, inherited from the mother. Both mutations were classified as pathogenic according to the ACMG 2015 guidelines, indicating a compound heterozygous inheritance pattern. The patient’s treatment regimen included phototherapy, which was initiated to address her jaundice upon admission. To support liver function and regulate gut activity, oral ursodeoxycholic acid (20 mg/kg/dose, twice a day) and probiotics were administered. Additionally, a postdischarge medication plan involving a low-dose regimen of phenobarbital (3.5 mg/kg/dose, twice a day) was implemented for 2 weeks.

**Results:**

During a 2-year follow-up after discharge, the infant’s bilirubin levels significantly decreased, and liver enzymes, including GGT, progressively normalized.

**Conclusion:**

This case report enhances the understanding of DJS in neonates by emphasizing the clinical ramifications of compound heterozygous mutations within the *ABCC2* gene and documenting the evolution of the disease. The gradual normalization of liver function tests suggests potential compensatory mechanisms in response to the genetic abnormalities in neonates with DJS. The correlation between the patient’s genetic profile of compound heterozygosity and her milder clinical phenotype warrants attention, suggesting that this specific genetic configuration may be associated with less severe manifestations of the disease. The necessity for long-term follow-up is highlighted, recognizing that intercurrent stress conditions could influence the hepatic profile and potentially exacerbate symptoms. Such sustained observation is crucial to further delineate the genomic and clinical landscape of DJS, offering opportunities to refine prognostic and therapeutic approaches.

## 1. Introduction

Dubin–Johnson syndrome (DJS) stands as an intriguing entity in the world of inherited liver disorders. Manifesting primarily as chronic, benign, intermittent jaundice, DJS is a consequence of impaired hepatic transport of conjugated bilirubin into bile. This defect culminates in the retention of bilirubin pigment in lysosomes, leading to the liver’s characteristic black appearance.^[1–3]^ Although the prevalence of DJS remains low in the global population, certain ethnic groups, particularly Iranian Jews and Japanese, show a higher increase in carrier rates.^[4,5]^ At the heart of DJS is the AR inheritance of mutations in the *ABCC2* gene, a key player in the transport of bilirubin and other organic anions from the liver into bile.^[6,7]^

Clinically, while DJS often reveals itself through mild jaundice from infancy or early childhood, the clinical manifestations and implications in newborns warrant deeper exploration.^[8,9]^ Early and precise diagnosis not only streamlines clinical management but also avoids potential complications arising from misdiagnosis with other hepatic conditions.^[10,11]^ This study presents a neonatal clinical perspective of the disease. Through this exploration, we may contribute to a better understanding of DJS’ infancy presentation and its developmental trajectory. For families navigating the challenges of a DJS diagnosis, early awareness and appropriate education can prove invaluable, offering clarity and direction in a potentially uncertain journey.

## 2. Ethical considerations and consent process

Written informed consent from the subjects’ guardians was obtained prior to admission. The study was approved by the Medical Ethics Committee of Anhui Provincial Children's Hospital (Anhui Hospital, Pediatric Hospital of Fudan University).

## 3. Patient initial presentation

The infant, a 19-day-old female, was brought to our facility due to observed abdominal distension. She was the second child, born to healthy and nonconsanguineous parents born at 36 weeks gestation via an uncomplicated vaginal delivery, there was no recorded history of asphyxia or hypoxia during the prenatal or perinatal periods. Concerns arose a day prior to her admission when family members noticed the infant’s abdominal bloating. An initial consultation at a local hospital revealed pale stools, leading to further investigations. A liver function test at the local hospital indicated elevated bilirubin levels, prompting her referral to our specialized institution. Throughout this period, the infant demonstrated a healthy appetite, feeding regularly on milk. There was no elevation in body temperature indicative of fever. However, detailed urinalysis showed her urine to be markedly dark yellow, consistent with the presence of increased conjugated bilirubin. Comprehensive laboratory evaluation upon admission also included chemical and physical stool analysis. Initially, her stools were pale yellow, but they gradually assumed a normal yellow coloration, the analysis of which did not indicate steatorrhea, thus helping to exclude certain gastrointestinal disorders that could contribute to malabsorption syndromes.

Upon examination, the infant displayed severe jaundice, which was apparent in both the skin and the sclera. The abdomen was notably distended, with visible veins apparent on the abdominal wall. Bowel sounds were absent, and there was no evidence of hepatosplenomegaly. Upon her admission, a series of imaging studies were conducted, including abdominal ultrasound and MRI of the liver and biliary system, none of which revealed any significant abnormalities or discrepancies (Table 1).

**Table 1:** Initial laboratory investigations on admission.

Laboratory investigations	Observed values	Normal values	Results
Total bilirubin	365.7 μmol/L		Elevated
Direct bilirubin	121.6 μmol/L		Elevated
Indirect bilirubin	244.1 μmol/L		Elevated
ALT	16 IU/L		Normal
AST	41 U/L		Normal
GGT	99 U/L		Elevated

ALT = alanine aminotransferase, AST = aspartate aminotransferase, GGT = gamma-glutamyl transferase.

The infant underwent phototherapy to address the jaundice when her bilirubin levels reached 15 mg/dL. Additionally, she received oral ursodeoxycholic acid (20 mg/kg/dose, twice a day) and probiotics to aid in gut regulation. Despite these interventions, the ursodeoxycholic acid demonstrated suboptimal efficacy in mitigating the abdominal distension, which did, however, gradually resolve following hepatic supportive and symptomatic treatments, with no recurrence of this symptom. Nonetheless, the direct bilirubin fraction exhibited a persistent increasing trend. Half a month postadmission, a liver stiffness measurement highlighted an average value of approximately 3.1 KPa, compared with normal liver stiffness values typically ranging between 2.0 and 2.5 KPa, pointing to alterations in liver consistency. Our decision not to utilize a gene panel specifically designed for neonatal cholestasis was based on the unique clinical manifestations and biochemical profile observed in this case, which suggested a broader spectrum of potential genetic disorders beyond the usual scope of cholestasis-related genes. Concurrently, blood tandem mass spectrometry for metabolic disorder screening revealed elevated levels of phenylalanine, necessitating further in-depth investigations via a trio whole-exome sequencing test. The test methods were as follows: (1) Mutation screening: Initially, with informed consent from the infant’s family, 2 mL of peripheral blood was drawn from the infant and her parents for high-throughput trio whole-exome sequencing. The inability to obtain samples from the infant’s elder brother posed a limitation in our study. This method comprehensively sequenced all protein-coding regions of a genome, furnishing exhaustive information on potential genetic mutations. (2) Genetic data analysis: After sequencing, we subjected the resultant data to an integrative, in-depth analysis, cross-referencing multiple databases and combining molecular biology annotations, genetic interpretations, and clinical characteristics, facilitated by a precision diagnostic cloud platform system designed for genetic disorders. Employing specialized genetic data analysis algorithms and adhering to the ACMG grading system, we classified hundreds of thousands of genetic variations. (3) Verification of suspected pathogenic mutations: Post high-throughput sequencing, we conducted PCR amplification on selected candidate loci with suspected pathogenic variations. These were then subject to Sanger sequencing, using the *ABI3730* sequencer, a revered method in mutation confirmation. Sequence analysis software was employed to interpret the results meticulously, confirming or ruling out pathogenic mutations. (4) Decision against invasive procedures: Crucially, our decision to prioritize genetic testing over cholangiography during the infant’s hospital stay was significantly influenced by specific clinical and radiological observations. The infant’s bowel movements were regular and lacked any signs of obstructive pathology, and the radiological findings did not indicate typical structural anomalies associated with cholestatic diseases. This clinical presentation, coupled with the distinctive radiological profile, suggested a metabolic or genetic etiology rather than a structural biliary disorder, thus steering our diagnostic approach toward genetic testing, which provided a noninvasive yet comprehensive method to explore potential hereditary or metabolic causes. Prior to the establishment of a definitive diagnosis, extensive screenings, including tests for common hereditary metabolic diseases such as PKU, were conducted. These tests confirmed the exclusion of these conditions as causal factors in the patient’s presentation. Moreover, we chose not to perform a liver biopsy despite the rare pathological findings of melanin deposits in newborn liver cells, considering it unnecessary in the face of advancing genetic testing technologies, which are progressively becoming more pivotal in diagnoses, negating the indispensability of liver biopsy as a sole diagnostic modality. Upon receiving the consequential genetic test results and a thorough assessment of the infant’s condition, the medical team decided to discharge the patient. In the subsequent postdischarge phase, the medical strategy involved the prescription of a regimen of phenobarbital (3.5 mg/kg/dose, twice a day) for 2 weeks, manifesting improved efficacy compared with the initial treatment, before its orderly discontinuation. To date, meticulous regular follow-ups with the patient have been sustained, ensuring close monitoring of her progress and prompt addressing of any emergent concerns or anomalies in her health status, underscoring the importance of continual surveillance in managing such conditions.

**Figure 1.**
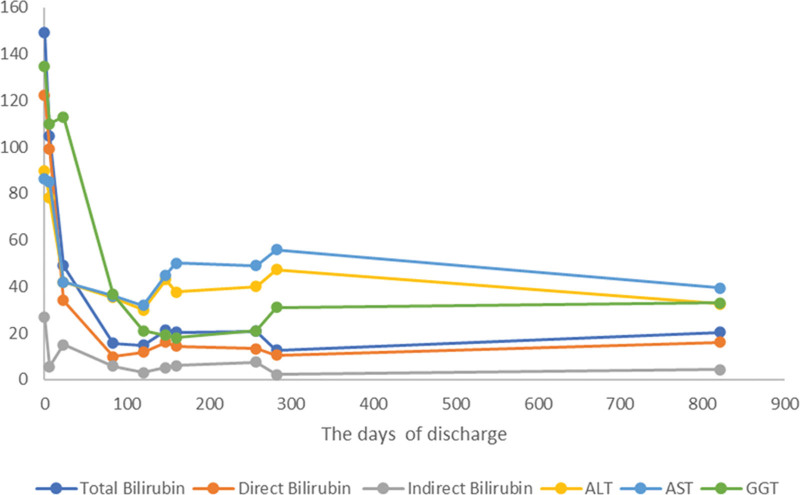
Physiological indicators after discharge.

During the 2-year follow-up after the patient’s discharge, there was a progressive improvement in the patient’s condition. The levels of total bilirubin, initially observed to be elevated, began a steady descent toward the normal range. Direct bilirubin also showed a decreasing trend, reflecting a positive response to treatments and interventions. The transaminases gradually returned to their standard values, further suggesting liver function restoration. Although the levels of GGT showed improvement, the pace of recovery was comparatively slower than the other indicators (Fig. 1). In terms of growth, the patient demonstrated a significant improvement in all anthropometric parameters. Her weight and height measurements were tracked along the 50th percentile for her age, indicating a normal growth trajectory. Head circumference, an essential parameter for neurological development in infants, was also within the normal range for her age group. This normal growth pattern was consistent with the improved clinical condition and adequate nutritional absorption. Additionally, gastrointestinal function and nutrient absorption were markedly regularized. There were no further episodes of steatorrhea or other signs of malabsorption, as evidenced by normal stools and absence of gastrointestinal discomfort. This was corroborated by improved appetite and dietary intake. Abdominal ultrasound data revealed a progressively normalizing liver texture and size. There were no signs of hepatic steatosis or other structural abnormalities. The gallbladder, bile ducts, and spleen appeared normal, with no evidence of dilation or other anomalies typically associated with liver diseases. These findings further supported the clinical and biochemical evidence of the liver’s functional recovery.

## 4. Identification of genetic variations

Upon comprehensive analysis of the gene sequencing results for the patient, our team was able to identify 2 significant genetic variations. (1) Insertion mutation: The patient displayed an insertion mutation located at *chr10:101606808-101606809*, designated as *c.4237(exon30)_c.4238(exon30)ins CT*. This mutation was inherited from the patient’s father. (2) Missense mutation: A missense mutation was observed at *chr10:101553697, labeled as c.517(exon5)G > A*. This particular mutation was inherited from the patient’s mother. Both of these genetic variations were critically evaluated in accordance with the ACMG Guidelines (2015).

**Figure 2.**
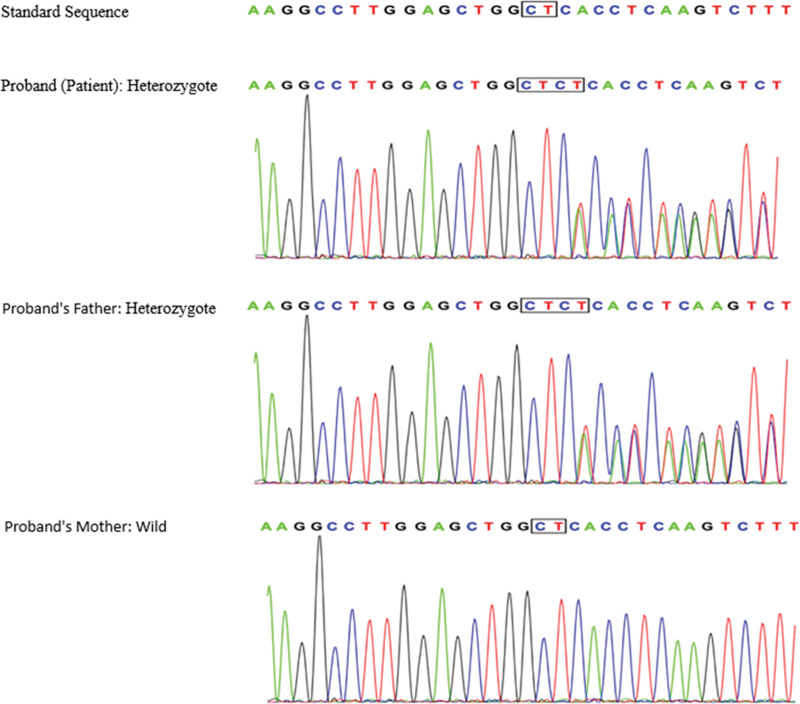
Mutation distribution in the family: *ABCC2:c.4237(exon30)_c.4238(exon30)ins CT*.

**Figure 3.**
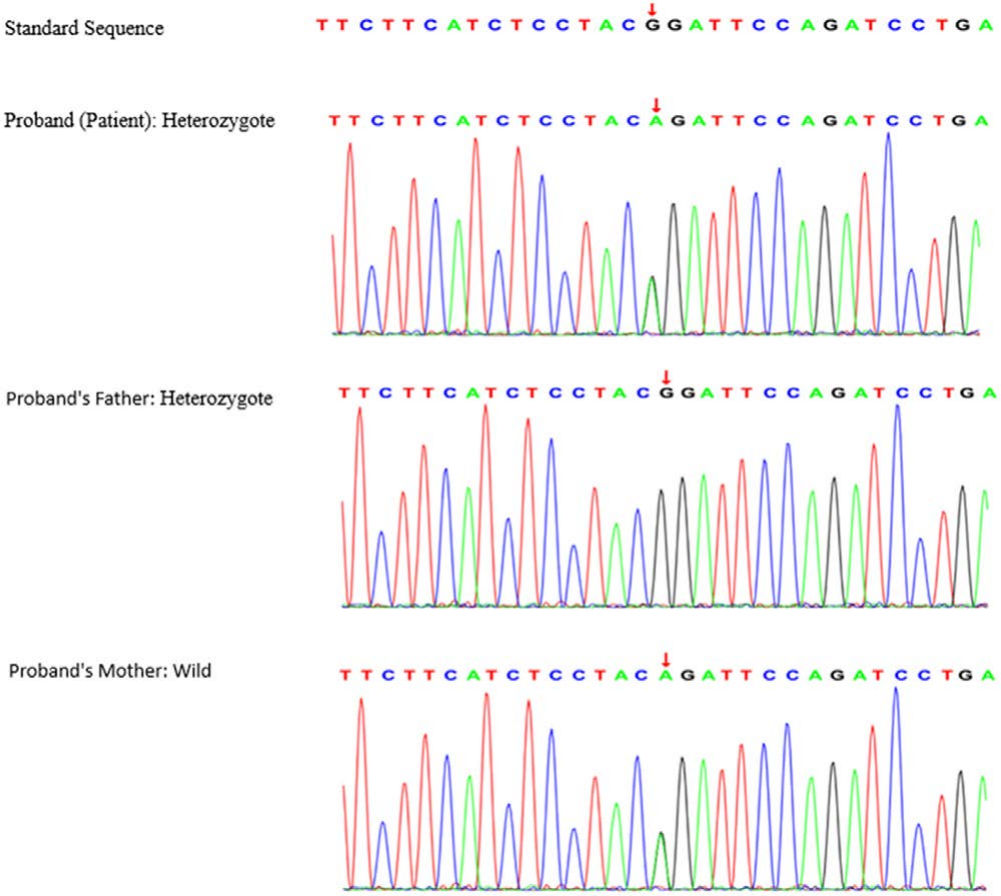
Mutation distribution in the family: *ABCC2:c.517(exon5)G > A*.

It was ascertained that the proband (the first-affected family member identified) is a heterozygote, aligning with the autosomal recessive (AR) compound heterozygous mechanism of disease inheritance. Additionally, the phenotypes observed in the proband and their family members aligned with typical AR inheritance patterns: the proband, inheriting 2 mutated alleles, exhibited the full clinical manifestation of the condition, while the parents, each carrying a single mutated allele, showed no clinical symptoms consistent with a heterozygous carrier status. This alignment of clinical presentations with genetic findings underscores the cosegregation of the observed phenotypes with the AR mode of transmission (Figs. 2 and 3).

## 5. Discussion

Dubin–Johnson syndrome is a rare inherited disorder predominantly characterized by a conjugated hyperbilirubinemia. The syndrome is attributed to mutations in the *ABCC2* gene, which impairs the hepatic secretion of conjugated bilirubin into bile.^[12,13]^

Inherited genetic disorders encompass a vast spectrum of variations, continually broadening our understanding of human genetics and challenging conventional clinical management strategies. The advent and integration of next-generation sequencing into clinical practice have been pivotal in elucidating complex clinical pictures that have often remained unresolved in the past. Next-generation sequencing plays a critical role in guiding the most appropriate management strategies and in providing invaluable insights for genetic counseling, thereby enhancing the precision and efficacy of both diagnosis and treatment in the realm of genetic disorders.^[14–16]^ Our highlighted case, where 2 unique mutations (*c.4237(exon30)_c.4238(exon30)ins CT* and *c.517(exon5)G > A*) on the chromosome 10 locus in the *ABCC2* gene were observed, is emblematic of compound intricacies. The coexistence of these mutations *c.4237(exon30)_c.4238(exon30)ins CT* and *c.517(exon5)G > A* presents a compelling platform to probe their combined clinical implications. The detailed analysis of these mutations is crucial: the insertion mutation potentially alters the reading frame, leading to a modified and possibly dysfunctional protein sequence downstream of the mutation.^[17]^ The missense mutation could result in a structural configuration alteration, affecting the normal function of the resultant protein.^[18]^ These uncharted mutations, especially 1 corresponding to a coding stop, are significant as they likely lead to premature termination and a truncated, possibly nonfunctional protein.^[19,20]^ Each mutation, independently, may disrupt the function of the *ABCC2* gene. However, when presented together, their combined effect could be magnified, potentially leading to an intensified clinical presentation. It begets the question of the functional repercussions of these mutations within the *ABCC2* gene. Investigations involving in vitro and in vivo models are pivotal to elucidate the precise biochemical and physiological impacts of these mutations, enriching our understanding of their role in the pathogenesis of DJS. The combined impact of these novel mutations may explain the persistence and severity of symptoms such as prolonged elevation in bilirubin levels.^[21,22]^ A systematic approach to collecting and analyzing data across diverse cases is vital to discerning potential patterns and correlations, enabling more accurate prediction of disease trajectories, and informing therapeutic strategies. Leveraging in vitro expression systems or animal models to mirror these mutations can offer invaluable insights into their phenotypic manifestations. Notably, 8 different *ABCC2* variants have been identified, ranging from more common ones like p.Arg768Trp and p.Arg100Ter to novel variants such as *p.Gly693Glu, p.Thr394Arg*, and *p.Asn718Ser*. Additionally, certain mutations like *c.2273G > T (Gly 758 val*), c.2443C > T (p.Arg815*), and *c.4179G > T (p.M1393I*) further exemplify the unique genetic landscape of this condition (Table 2).^[5,10,23,24]^ Our case emphasizes the critical importance of correlating genotype with phenotype. It suggests that compound heterozygosity might be associated with a milder clinical phenotype and more favorable outcomes, potentially indicative of an inherent resilience within the neonatal system. The observed amelioration in bilirubin and liver enzyme levels over the 2-year postdischarge period in our patient provides an optimistic perspective on the prognosis of such genetic perturbations. This pattern of recovery could indicate a potential intrinsic ability of the neonatal system to partially offset or adapt to the detrimental effects of these genetic anomalies. It is plausible that while the body manages to find alternative mechanisms or workarounds for other disrupted pathways due to the mutations, the bile secretion pathway, indicated by GGT levels, faces prolonged recovery. This could be due to lasting structural or functional impediments in the bile ducts or a more direct consequence of the mutations’ effect on bile transporters.

**Table 2:** Recently study of ABCC2 mutations in DJS.

Author	Number of patients	ABCC2 mutations
Corpechot, C	32	High diversity of point mutations and copy number variations
Kim, K	6	8 different ABCC2 variants, including common ones like p.Arg768Trp and p.Arg100Ter, and novel variants p.Gly693Glu, p.Thr394Arg, p.Asn718Ser
Al-Hussaini, A	11	Common ABCC2 gene variant: c.2273G > T (Gly 758 val)
Khabou, B	–	Novel ABCC2 mutation: c.2443C > T (p.Arg815*) and c.4179G > T (p.M1393I)

## 6. Conclusions

The genetic underpinnings of this case have unraveled noteworthy mutations that provide insights into the clinical manifestations of DJS. The ability of the neonatal system to demonstrate significant recovery, despite such genetic challenges, speaks to its intrinsic adaptive capacities. However, the differential recovery patterns, particularly the prolonged normalization of GGT levels, highlight the persistent challenges in the hepatobiliary system and emphasize the need for a more comprehensive understanding of the long-term effects of genetic mutations. In considering these dynamics, it is also important to acknowledge the potential role of epigenetic and environmental factors in modulating the patient’s phenotype, which could further influence the long-term clinical evolution and adaptive responses.^[25,26]^ We acknowledge this potential course and have planned to continue our follow-up with the girl to monitor any developments or changes in her condition closely. This case not only adds to the expanding repository of genetic studies but also accentuates the importance of tailored medical approaches, grounded in a deep appreciation of both the genetic and adaptive nuances of individual patients, with consideration of the long-term clinical evolution, adaptative responses, and the potential influence of epigenetic and environmental factors in the face of genetic aberrations.

## Author contributions

**Conceptualization:** Yanmin Zhang, Wei Zuo.

**Data curation:** Yanmin Zhang, Wei Zuo, Wei Gao.

**Formal analysis:** Yanmin Zhang, Wei Zuo, Wei Gao.

**Investigation:** Yanmin Zhang, Wei Zuo, Wei Gao.

**Validation:** Yanmin Zhang, Wei Zuo, Wei Gao.

**Writing – original draft:** Yanmin Zhang.

**Writing – review & editing:** Wei Zuo.
